# Effect of Mesalamine and Prednisolone on TNBS Experimental Colitis, following Various Doses of Orally Administered Iron

**DOI:** 10.1155/2014/648535

**Published:** 2014-05-04

**Authors:** John K. Triantafillidis, Georgia Douvi, George Agrogiannis, Efstratios Patsouris, Aristofanis Gikas, Apostolos E. Papalois

**Affiliations:** ^1^Inflammatory Bowel Disease Unit, IASO General Hospital, 264 Mesogeion Avenue, Holargos, 15562 Athens, Greece; ^2^1st Department of Pathology, University of Athens, School of Medicine, 75 Mikras Asias, 115 27 Athens, Greece; ^3^Health Center of Kalivia, Attiki, 1st Klm Kalivion Avenue, 19010 Kalivia, Greece; ^4^Experimental and Research Center, ELPEN Pharma, 95 Marathonos Avenue, Pikermi, 19009 Athens, Greece

## Abstract

*Background*. Experimental data suggest that oral iron (I.) supplementation can worsen colitis in animals. * Aim*. To investigate the influence of various concentrations of orally administered I. in normal gut mucosa and mucosa of animals with TNBS colitis, as well as the influence of Mesalamine (M.) and Prednisolone (P.) on the severity of TNBS colitis following orally administered I. * Methods and Materials*. 156 Wistar rats were allocated into 10 groups. Colitis was induced by TNBS. On the 8th day, all animals were euthanatized. Activity of colitis and extent of tissue damage were assessed histologically. The levels of tissue tumor necrosis factor-**α** (t-TNF-**α**) and tissue malondialdehyde (t-MDA) were estimated in all animal groups. * Results*. Moderate and high I. supplementation induced inflammation in the healthy colon and increased the activity of the experimentally induced TNBS colitis. Administration of M. on TNBS colitis following moderate iron supplementation (0.3 g/Kg diet) resulted in a significant improvement in the overall histological score as well as in two individual histological parameters. M. administration, however, did not significantly reduce the t-TNF-**α** levels (17.67 ± 4.92 versus 14.58 ± 5.71, *P* = 0.102), although it significantly reduced the t-MDA levels (5.79 ± 1.55 versus 3.67 ± 1.39, *P* = 0.000). Administration of M. on TNBS colitis following high iron supplementation (3.0 g/Kg diet) did not improve the overall histological score and the individual histological parameters, neither reduced the levels of t-TNF-**α** (16.57  ± 5.61 versus 14.65 ± 3.88, *P* = 0.296). However, M. significantly reduced the t-MDA levels (5.99 ± 1.37 versus 4.04 ± 1.41, *P* = 0.000). Administration of P. on TNBS colitis after moderate iron supplementation resulted in a significant improvement in the overall histological score as well as in three individual histological parameters. P. also resulted in a significant reduction in the t-TNF-**α** levels (17.67 ± 4.92 versus 12.64 ± 3.97, *P* = 0.003) and the t-MDA levels (5.79 ± 1.54 versus 3.47 ± 1.21, *P* = 0.001). Administration of P on TNBS colitis after high I. supplementation resulted in a significant improvement of the overall histological score and three individual histological parameters and significantly reduced the levels of t-TNF-**α** (16.6 ± 5.6 versus 11.85 ± 1.3, *P* = 0.001). * Conclusion*. I. can induce colonic inflammation and aggravate TNBS colitis. M. and P. can significantly improve the inflammatory process in the colonic mucosa in TNBS colitis aggravated by orally administered I. P. has a stable anti-TNF-**α** effect. These findings suggest that the harmful.

## 1. Introduction

Patients with long-standing inflammatory bowel disease (IBD) frequently develop anaemia, the pathogenesis of which is attributed to the underlying chronic disease, the iron deficiency, or both. Iron depletion is usually attributed to increased loss, impaired absorption from the gut, or both [1]. As a consequence, most patients with IBD are advised to consume foods rich in iron and proteins as well as to receive orally iron for a long period of time [2]. It is well established that the orally administered iron can react in the inflamed colon with superoxide (O_2−_) and hydrogen peroxide (H_2_O_2_), thus producing free hydroxyl radical (OH) that can induce oxidative stress. The latter could further aggravate the tissue damage and amplify the degree of inflammation. Experimental data suggest that oral iron supplementation can induce colitis in animals and amplify the inflammatory response [3–8].

In patients with IBD, iron derived from degradation of heme in the inflamed bowel mucosa could worsen the degree of inflammation via the same mechanism. The oxidative stress generated by iron supplementation could worsen the extension and propagation of crypt abscesses [9]. The harmful effect of I. is further supported by data showing that deferiprone protects against the development of colitis, by decreasing the production of iron-dependent oxygen-free radicals [10]. Taking into account that the antioxidant defence system is impaired in patients with IBD, it seems that the inflammatory process perpetuates via the creation of this vicious circle [11, 12]. Clinical data suggest that administration of ferrous fumarate in patients with I. deficiency might deteriorate plasma antioxidant status and worsen the clinical symptoms of patients with active Crohn's disease [13, 14], although others suggest that the orally administered I. is well tolerated by most IBD patients [15, 16]. DNA damage caused by oxidative stress is a major contributor to colorectal cancer development in IBD [17, 18].

Data referring to the effect of orally administered I. in normal and experimentally inflamed bowel mucosa are scarce. Almost no data referring to the influence of mesalamine (M.) on experimental colitis aggravated by orally administered I. are available, and so far, no data referring to the influence of prednisolone (P.) exist.

The purpose of this study was to investigate (a) the effect of orally administered I. on the degree of inflammation and oxidative stress in the large bowel of normal rats, (b) the effect of orally administered I. on the degree of colonic inflammation in TNBS colitis, and (c) the influence of M. and P. on the degree of colonic inflammation in rats with TNBS colitis concurrently receiving moderate and high I. doses.

## 2. Materials and Methods

The experimental procedures described below were approved by the Animal Care Committee according to the European Union Act and Greek Law 160 A-64 May 1991.

### 2.1. Experimental Animals

Adult male Wistar rats weighing 200–240 g were allowed to adapt to our laboratory conditions 1 wk prior to the experiment. They were housed individually in cages at a constant temperature (22°C) and in a 12 h d/night cycle with free access to food and water. A total number of 156 rats were used.

### 2.2. Induction of Experimental Colitis

Distal colitis was induced by intracolonic installation of 25 mg of TNBS dissolved in 0.25 mL of 50% ethanol. The solution was injected into the colon 8 cm proximal to the anus with a PE-50 cannula. In order to ensure that TNBS-ethanol solution was not immediately expelled by the rat, the cannula was left in place for 15 s prior to its removal. On day 8, all rats were anaesthetized by intraperitoneal administration of sodium pentobarbital (50 mg/kg) and sacrificed by cardiac puncture. Blood was then drawn into an ethylenediaminetetraacetic acid- (EDTA-) containing vacutainer, centrifuged at 3000 g for 10 min and plasma was stored at −80°C.

### 2.3. Iron Supplementation

A regular diet containing 270 mg of iron/Kg diet (0.027%) was used in all groups. However, iron supplemented groups received either 3,000 mg of iron/Kg diet (iron 0.3%) or 30,000 mg of iron/Kg diet (iron 3%) in the form of water solution [4]. It must be stressed that the iron 3% diet is 100-fold the regular diet, while the iron 0.3% (10-fold the regular diet) is quite similar to the amount of iron used therapeutically in the clinical practice.

In order to ensure that the animals received the amount of iron according to the design of the experiment, iron was administered orally with the help of a plastic syringe, up to the last day of the experiment.

### 2.4. Drug Administration

M. and P. were orally administered every day via a mouth catheter in a dose of 30 and 1 mg/Kg/day, respectively.

### 2.5. Experimental Groups

The experimental animals were randomly allocated into 10 groups according to the dose of iron (Fe) and the kind of drug administered. Group 1: Fe 0.027% (normal diet), group 2: Fe 0.3 g diet, group 3: Fe 3.0 g diet, group 4: TNBS colitis plus Fe 0.027%, group 5: TNBS colitis plus Fe 0.3 g diet, group 6: TNBS colitis plus Fe 3 g diet, group 7: TNBS colitis plus Fe 0.3 g diet plus mesalamine, group 8: TNBS colitis plus Fe 3 g diet plus mesalamine, group 9: TNBS colitis plus Fe 0.3 g diet plus prednisolone, and group 10: TNBS colitis plus Fe 3 g diet plus prednisolone.  Table 1 shows the characteristics of these experimental groups.

### 2.6. Histology

At the end of the experiment, the colon was removed, opened, and cut longitudinally for histological examination and tissue measurements, respectively. Specimens were fixed in 10% buffered formalin and embedded in paraffin blocks (3-4 blocks for each case). Hematoxylin-eosin stained sections were, then, examined by the pathologist participating in the study, who was completely aware of the origin of specimens. Activity of colitis and extent of tissue damage were assessed histologically by estimating the following seven parameters: degree of inflammation, infiltration by eosinophils, infiltration by neutrophils, presence of erosions, presence of epithelial damage, presence of gland distortion, and presence of edema. Lesions were graded according to the absence (0) or the presence of a specific one (1 to 3). Then, on the basis of the degree of lesions, an overall histological score for each animal was created. The severity of colitis was estimated according to Geboes et al. [19]. It must be stressed, however, that the severity of this kind of colitis includes “quality” characteristics, as well. For example, an abundance of eosinophils could be found in one animal, while in another a small number of eosinophils and neutrophils could be observed. The overall score could be the same in both groups; however, in the second case the grade would be higher. Because eosinophils can be found in the bowel mucosa of normal animals, we graded as “eosinophilic infiltration” only if an obvious increase in the number of eosinophils in the mucosa was present. Finally, we noticed that in experimental animals with severe colitis graded 5.2 or 5.3 according to Geboes et al. [19] the findings were highly focal with the surrounding mucosa being almost normal.

### 2.7. Tissue TNF-*α* (t-TNF-*α*) Estimation

TNF-*α* was determined after tissue homogenization by ELISA. In order to avoid errors in the interpretation of results, a specific rat antibody was used (antirat, DIACLONE Research) instead of human antibody against TNF-*α*.

### 2.8. Tissue Malondialdehyde (t-MDA) Estimation

The MDA measurement was performed as it was previously described [20] and it is based on the reaction of a chromogenic reagent,* N*-methyl-2-phenylindole (MPI), with MDA at 45°C. One molecule of MDA reacted with two molecules of MPI to yield a stable chromophore with maximum absorbance at 586 nm. The reagents used included Reagent MPI, 10.3 mmol/L* N*-methyl-2-phenylindole in acetonitrile, MDA standard, 10 mmol/L 1,1,3,3-tetramethoxypropane in 20 mmol/L Tris-HCl, 500 mmol/L butylated hydroxytoluene, in acetonitrile, 20 mmol/L Tris buffer pH 7.4, 0.9% NaCl, 37% (12 mol/L) HCl, methanol, HPLC grade, acetonitrile and HPLC grade. Before the procedure, three volumes of the MPI reagent were diluted with one volume of 100% methanol. Tissue samples were rinsed with ice-cold isotonic saline before homogenization which was carried out using Tris buffer 20 mmol/L pH 7.4 and an ULTRA-TURRAX (IKA-Labortecnik) blender. One millilitre buffer was used for 0.1 g of tissue. Ten millilitres of 500 mmol/L BHT was added to 1 mL of tissue homogenate to prevent sample oxidation. The homogenate was centrifuged at 3,000 r/min at 4°C for 10 min. Then, 0.2 mL of sample (plasma or supernatant of tissue homogenate) and 0.65 mL of diluted MPI reagent were added to a polypropylene microcentrifuge tube. The mixture was vortexed and then 0.15 mL of 12 mol/L HCl was added. Tubes were incubated at 45°C for 60 min and centrifuged at 6,000 r/min for 15 min. Then, 0.8 mL of the supernatant was measured at 586 nm. MDA standards for the standard curve were made by dilutions of the stock 10 mm TMOP solution. The final concentrations were 2.08, 4.16, 8.33, 12.5, and 16.66 *μ*mol/L and the assay procedure was followed as for the samples. The absorbance was 0.059, 0.124, 0.264, 0.4, and 0.545, respectively.

### 2.9. Statistical Analysis

All results obtained are expressed as the mean ± SD as indicated. The significance of differences was calculated either by the Pearson chi-squared test, Student's two-tailed *t* test, or one-way ANOVA with Tukey's post hoc test. A difference between groups was considered significant at *P* < 0.05 (2-tailed). Computations were done using the statistical package SPSS (version 17.0).

## 3. Results

### 3.1. Effect of Iron Supplementation on Colonic Mucosa of Experimental Animals without Colitis (Groups 1, 2, and 3)

At the end of the experiment, 155 animals were alive; one death was noticed in group 10.

*(1) Histology.* As it is shown in Table 2, there was a statistically highly significant difference among the 3 groups concerning overall lesions as determined by one-way ANOVA (*P* = 0.000). A Tukey post hoc test revealed that the mean value of lesions was significantly higher in groups 2 (*P* = 0.002) and 3 (*P* = 0.000) compared to group 1. There were no significant differences between group 2 and group 3 (*P* = 0.073). Significant differences in some individual histological parameters among the three groups were noticed, namely, inflammatory infiltration (8.3% versus 16.7% versus 50%, *P* = 0.045) and eosinophilic infiltration (8.3% versus 100% versus 100%, *P* = 0.000).

*(2) t-TNF-*α* Levels.* As the levels of t-TNF-*α* in group 1 were not detectable, calculations were possible only between groups 2 and 3. No significant differences in the t-TNF-*α* levels between groups 2 and 3 were noticed (*P* = 0.66).

*(3) t-MDA Levels.* There was a statistically significant difference among the 3 groups concerning t-MDA levels as determined by one-way ANOVA (*P* = 0.014). A Tukey post hoc test revealed that the mean value of t-MDA was significantly higher in group 3 compared to group 1 (*P* = 0.018).

### 3.2. Effect of Iron Supplementation on Colonic Mucosa after Induction of TNBS Colitis (Groups 4, 5, and 6)

*(1) Histology.* As it is shown in Table 3, significant differences among the 3 groups concerning the overall histological score were noticed. A Tukey post hoc test revealed significant differences between group 4 versus group 5 (*P* = 0.000) and group 4 versus group 6 (*P* = 0.007), but not between group 5 versus group 6 (*P* = 0.526). I. supplementation worsened individual histological parameters (oedema; 12.5% versus 79.2% versus 87.5%, *P* = 0.0001).

*(2) t*-*TNF-*α* Levels.* No significant differences in the t-TNF-*α* levels among the 3 groups were noticed (Table 3). This was evident in the Tukey post hoc test (group 4 versus group 5: *P* = 0.089, group 4 versus group 6: *P* = 0.327 and group 5 versus group 6: *P* = 0.764).

*(3) t-MDA Levels.* Significant differences in the t-MDA levels among the 3 groups were noticed (Table 3). A Tukey post hoc test revealed significant differences between group 4 versus group 5 (*P* = 0.000) and group 4 versus group 6 (*P* = 0.000), but not between group 5 versus group 6 (*P* = 0.856).

### 3.3. Effect of I. Supplementation on TNBS Colitis following Treatment with Mesalamine

#### 3.3.1. Effect of Mesalamine after Iron Supplementation of 0.3 g/Kg Diet

*(1) Histology.* Table 4 shows the effect of M. administration on TNBS colitis after I. supplementation of 0.3 g/Kg diet. There were significant reductions in the overall histological score, inflammatory and eosinophilic infiltration, and edema.

**Figure 1:**
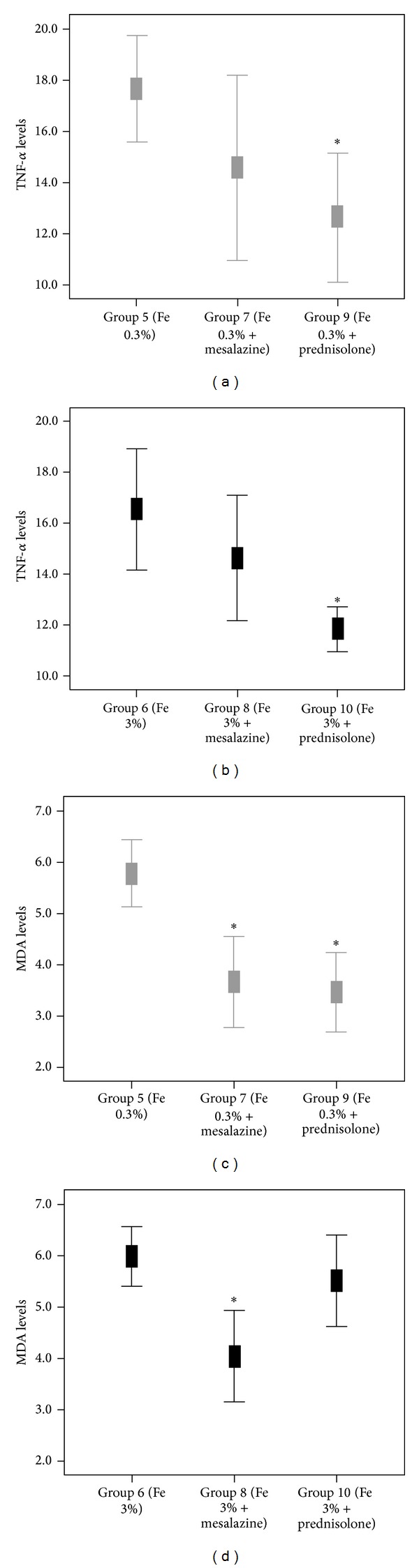
Mean values of TNF-*α* (pg/mL) and MDA (*μ*mol/L) levels in various groups of animal models with experimental colitis that received moderate ((a) and (c)) and high ((b) and (d)) concentrated iron supplements. (*P ≤ 0.003, in comparison with respective referent group 5 or 6).

*(2) t-TNF-*α* Levels.* M. administration resulted in a nonsignificant reduction in the t-TNF-*α* levels in group 5 compared to group 7 (17.67 ± 4.92 versus 14.58 ± 5.71, *P* = 0.102) (Figure 1(a)).

*(3) t-MDA Levels.* M. administration resulted in a significant reduction in the t-MDA levels in group 5 compared to group 7 (5.79 ± 1.55 versus 3.67 ± 1.39, *P* = 0.000) (Figure 1(c)).

#### 3.3.2. Effect of Mesalamine after Iron Supplementation of 3.0 g/Kg Diet

*(1) Histology.* Table 4 shows the effect of M. administration in experimental colitis after I. supplementation of 3.0 g/Kg diet. Comparisons between groups 6 and 8 revealed a significant increase in the overall histological score and in inflammatory infiltration. There were no significant differences concerning eosinophilic infiltration and edema.

*(2) t-TNF-*α* Levels.* M. administration resulted in a nonsignificant reduction in the t-TNF-*α* levels (16.57 ± 5.61 versus 14.65 ± 3.88, *P* = 0.296) (Figure 1(b)).

*(3) t-MDA Levels.* M. administration resulted in a significant reduction in the t-MDA levels in group 6 compared to group 8 (5.99 ± 1.37 versus 4.04 ± 1.41, *P* = 0.000) (Figure 1(d)).

### 3.4. Effect of Iron Supplementation on TNBS Colitis following Treatment with Prednisolone 

#### 3.4.1. Effect of Prednisolone on TNBS Colitis after Iron Supplementation of 0.3 g/Kg Diet

*(1) Histology.* Table 5 shows the effect of prednisolone administration on TNBS colitis after I. administration 0.3 g/Kg diet. A significant improvement was noticed in the overall histological score as well as in three individual histological parameters, namely, inflammatory and eosinophilic infiltration and edema.

*(2) t-TNF-*α* Levels.* P. administration resulted in a significant reduction in the t-TNF-*α* levels in group 5 compared to group 9 (17.67 ± 4.92 versus 12.64 ± 3.97, *P* = 0.003) (Figure 1(a)).

*(3) t-MDA Levels.* P. administration resulted in a significant reduction in t-MDA levels in group 5 compared to group 9 (5.79 ± 1.54 versus 3.47 ± 1.21, *P* = 0.001) (Figure 1(c)).

#### 3.4.2. Effect of Prednisolone on TNBS Colitis after Iron Supplementation of 3.0 g/Kg Diet

*(1) Histology.* Table 5 shows the effect of prednisolone administration on experimental colitis after iron administration 3.0 g/d. Comparisons between groups 6 and 10 revealed a significant reduction in the overall histological score, as well as in three histological parameters, namely, inflammatory infiltration, eosinophilic infiltration, and edema.

*(2) t-TNF-*α* Levels.* P. administration resulted in a significant reduction in t-TNF-*α* levels (16.6 ± 5.6 versus 11.85 ± 1.3, *P* = 0.001) (Figure 1(b)).

*(3) t-MDA Levels.* P. administration resulted in a nonsignificant reduction in t-MDA levels in group 6 compared to group 10 (5.99 ± 1.37 versus 5.51 ± 1.31, *P* = 0.339) (Figure 1(d)).

## 4. Discussion

The present study was conducted in order to see if the oral administration of I. could be harmful in healthy animals as well as animals with TNBS colitis and in that case, if the administration of mesalamine (M) or prednisolone (P) could reduce or ameliorate the degree of experimental colitis aggravated by I. administration.

The results showed that oral I. supplementation, at either moderate or high doses, can induce inflammation in the large bowel mucosa of healthy rats. This conclusion was based on the significant worsening of both, the overall histological score and individual histological parameters (inflammatory and eosinophilic infiltration) in groups with high I. consumption compared with healthy animals on normal I. consumption. t-TNF-*α* levels were found to be increased in the groups with high I. diet compared with animals on normal I. diet. In addition, high I. supplementation induced oxidative stress in these groups compared with rats being on normal food I.

It was also shown that both, moderate and high I. supplementation, significantly worsen the histological score in TNBS colitis, although differences between groups with moderate or high I. supplementation did not reach statistical significance. I. supplementation, either moderate or high, induced oxidative stress. No differences in the levels of t-TNF-*α* were noticed, suggesting that I. supplementation does not increase the production of TNF-*α* by the macrophages in this model of colitis.

The administration of M. significantly reduced the grade of histological score and individual histological parameters in rats with TNBS colitis being on moderate but not high I. supplementation compared to groups with TNBS colitis and moderate or high I. supplementation. M. caused nonsignificant reduction in t-TNF-*α* levels in both moderate and high I. supplementation. M. also reduced the grade of oxidative stress as it was evident by the reduction of t-MDA levels in both moderate and high I. supplementation.

It was also found that P. significantly improved the overall histological score and individual histological parameters in animals with both moderate and high I. supplementation. P. significantly reduced the t-TNF-*α* levels in both moderate and high I. supplementation suggesting a stable anti-TNF-*α* effect of the drug. P. also resulted in a significant reduction in oxidative stress in moderate but not high I. supplementation. We are not able to offer a good explanation why P. reduced the levels of oxidative stress in the group with moderate I. supplementation and not in the group with high I. supplementation. One possible explanation might be related to the “amount” of oxidative stress appearing in the two groups. Probably higher doses of P. could result in a greater reduction in the oxidative stress but this remains a speculation.

TNBS colitis is considered to be one of the most appropriate and successful models of experimental colitis because the inflammatory processes have a relative long duration and the histological lesions resemble those of human IBD [21, 22]. In this model of colitis, the colonic mucosa shows an activation of arachidonic acid pathway and inducible nitric oxide synthase [22]. Mucosal TNF-*α* is necessary for the initiation and perpetuation of TNBS colitis, since TNF-*α*-deficient mice are resistant to TNBS, and the colitis is extremely severe in mice that overexpress TNF-*α* [23, 24]. In ulcerative colitis patients, TNF-*α* is highly expressed [25]. Reactive oxygen species enhance the production of TNF-*α* and activate NF-*κ*B which subsequently enhances the production of TNF-*α*.

Oxidative stress has been considered as an important mechanism underlying the pathophysiology of IBD. A sustained production of reactive oxygen species during colonic inflammation can overwhelm the antioxidant defense system [26], which can be further impaired by iron supplementation. In patients with active IBD, superoxide and hydrogen peroxide free radicals interact with iron of the bowel lumen to yield the highly reactive hydroxyl radicals which enhance the intestinal inflammation by increasing intestinal permeability and the recruitment and activation of neutrophils.

The harmful effect of oral I. administration in inducing bowel mucosa inflammation has been reported in both, human and experimental animals. Without exception, all studies agree that I. supplementation results in intestinal inflammation in both healthy and experimentally inflamed intestinal mucosa. Carrier et al. demonstrated that iron 0.3% and 3% increase both, the activity of DSS experimental colitis and the level of oxidative stress [4]. Erichsen et al. described that even low dose of oral ferrous I. could enhance intestinal inflammation in DSS colitis in rats [5]. Reifen et al. found that I. supplementation can amplify the inflammatory response and the mucosal damage in experimental colitis [27]. Mucosal biopsies from patients with ulcerative colitis have increased reactive oxygen intermediates and reduced the levels of copper and zinc, two well-known cofactors for the endogenous antioxidant system [28]. Lipid oxidation is significantly harmful for the bowel mucosa leading to cellular necrosis. The harmful effect of I. supplementation can be reduced by the addition of vitamin E [3]. After oral supplementation, I. deposition can be only seen at the mucosa, while parenteral I. can result in I. deposition throughout the intestinal wall. The activation of NF-*κ*B by iron-generated free radicals could be important [29]. Epidemiological data suggested that the risk of appearance of IBD could be related to high I. diet content. In a relevant study, there was a 21% increase in the risk of development of IBD if the I. content of the drinking water increased by 0.1 mg/L. The mechanism is probably related to the induction of oxidative stress subsequently causing inflammation and cell mutations [30]. The harmful effect of I. on the colonic mucosa is further supported by experiments showing that rats assigned to iron-deprived diet or to a chelation substance preceding the induction of colitis have less diarrhoea and loss of weight compared to untreated rats [6, 31–33]. Finally, I. sulphate free diet in combination with systemic I. repletion ameliorates ileitis in a murine model of Crohn's disease [34].

The harmful effect of I. supplementation on the colonic mucosa is further supported by clinical data. In a relevant study, the administration of I. in healthy volunteers resulted in a significant increase in the fecal free radical production [35]. Iron can be found in the colonic mucosa, thus increasing the level of oxidative stress [36]. In a clinical trial, it was noticed that the administration of ferrous sulphate in patients with Crohn's disease can increase the levels of plasma MDA [37]. However, others suggest that orally administered I. is well tolerated by most IBD patients and does not exacerbate their symptoms [15].

Because I.-deficient anaemia represents a major clinical problem in IBD patients, physicians usually suggest the peros administration of I. Our results showed that M. could ameliorate or reduce the harmful effect of orally administered I. So far, two studies have been published concerning M. In the first one, Reifen et al. described that simultaneous administration of M. attenuated the harmful effect of I. in experimental colitis [9]. In the second study, Axelsson et al. noticed that both sulfasalazine (100 mg/Kg/d) and olsalazine (50 mg/Kg/d) significantly improved some clinical parameters and ameliorated the DSS-induced intestinal inflammation of rats with DSS colitis [38]. Our results also showed that P. exerts a significantly beneficial effect on TNBS-induced colitis aggravated by moderate or high I. supplementation. This widely used drug can reduce both, the levels of oxidative stress and the t-TNF-*α* levels, a finding not reported so far.

*In Conclusion* we demonstrated that orally administered I. can induce inflammation in the colon of healthy rats and aggravate colonic injury in TNBS-induced colitis. We also demonstrated that the two most commonly used drugs in the treatment of IBD, namely, M. and P. can ameliorate or significantly reduce the inflammatory process of the colonic mucosa induced by I. supplementation in TNBS colitis. Because I. supplementation is regularly used in the treatment of iron-deficient anaemia of patients with IBD, our findings might be clinically relevant. We suggest the concurrent administration of M. in patients with active or inactive IBD receiving orally I. The results of this study must be confirmed in patients with active or inactive IBD.

## Figures and Tables

**Table 1:** Animal groups and drug administered.

Group	TNBS colitis	Normal Iron content	Iron content of 0.3 g	Iron content of 3 g	Mesalamine	Prednisolone	Number of living animals at the end of the experiment
1		#					12/12
2			##				12/12
3				∗∗			12/12
4	∗	#					24/24
5	∗		##				24/24
6	∗			∗∗			24/24
7	∗		##		###		12/12
8	∗			∗∗	###		12/12
9	∗		##			∗∗∗	12/12
10	∗			∗∗		∗∗∗	11/12

**Table 2:** Overall histological score, and t-TNF-*α* and t-MDA levels in groups 1, 2, and 3.

	Group 1	Group 2	Group 3	*P*
Overall	0.25 ± 0.62	1.33 ± 0.65	2.00 ± 0.85	0.000
t-TNF-*α* ^a^	0.00	12.2 ± 3.53	12.8 ± 3.17	
t-MDA	2.49 ± 0.54	3.52 ± 1.93	5.36 ± 2.35	0.014

^a^group 2 versus 3, *P* = 0.66.

**Table 3:** Overall histological score, and t-TNF-*α* and t-MDA levels in groups 4, 5, and 6.

	Group 4	Group 5	Group 6	*P*
Overall	2.58 ± 0.58	4.21 ± 1.77	3.79 ± 1.35	0.000
t-TNF-*α*	14.3 ± 5.77	17.7 ± 4.92	16.6 ± 5.55	0.10
t-MDA	3.05 ± 0.83	5.79 ± 1.54	5.99 ± 1.36	0.000

**Table 4:** Differences in histological parameters between group 5 (TNBS + Fe 0.3%) versus group 7 (TNBS + Fe 0.3% + mesalamine) and group 6 (TNBS + Fe 3%) versus group 8 (TNBS + Fe 3% + mesalamine).

Parameter	Group 5 versus 7	*P* value	Group 6 versus 8	*P* value
Overall histology	4.21 ± 1.77 versus 2.00 ± 0.74	0.000	3.79 ± 1.35 versus 4.67 ± 0.65	0.013
Inflammatory infiltration	1.58 ± 0.72 versus 0.67 ± 0.65	0.001	1.37 ± 0.71 versus 2.42 ± 0.51	0.000
Eosinophilic infiltration	1.54 ± 0.72 versus 1.00 ± 0.00	0.001	1.33 ± 0.48 versus 1.08 ± 0.29	0.061
Edema	1.08 ± 0.72 versus 0.33 ± 0.49	0.003	1.08 ± 0.58 versus 1.17 ± 0.39	0.658

**Table 5:** Differences in histological parameters between group 5 (TNBS + Fe 0.3%) versus group 9 (TNBS + Fe 0.3% + prednisolone) and group 6 (TNBS + Fe 3%) versus group 10 (TNBS + Fe 3% + prednisolone).

Parameter	Group 5 versus 9	*P* value	Group 6 versus 10	*P* value
Overall histology	4.21 ± 1.77 versus 2.08 ± 0.79	0.000	3.79 ± 1.35 versus 2.18 ± 1.08	0.001
Inflammatory infiltration	1.58 ± 0.72 versus 0.50 ± 0.67	0.000	1.37 ± 0.71 versus 0.54 ± 0.69	0.003
Eosinophilic infiltration	1.54 ± 0.72 versus 1.00 ± 0.00	0.001	1.33 ± 0.48 versus 1.00 ± 0.00	0.003
Edema	1.08 ± 0.72 versus 0.58 ± 0.51	0.039	1.08 ± 0.58 versus 0.64 ± 0.67	0.053
